# Dual Regulation of Cadmium-Induced Apoptosis by mTORC1 through Selective Induction of IRE1 Branches in Unfolded Protein Response

**DOI:** 10.1371/journal.pone.0064344

**Published:** 2013-05-16

**Authors:** Hironori Kato, Ryohei Katoh, Masanori Kitamura

**Affiliations:** 1 Department of Molecular Signaling, Interdisciplinary Graduate School of Medicine and Engineering, University of Yamanashi, Chuo, Yamanashi, Japan; 2 Department of Pathology, Interdisciplinary Graduate School of Medicine and Engineering, University of Yamanashi, Yamanashi, Japan; Kinki University School of Pharmaceutical Sciences, Japan

## Abstract

Cadmium (Cd) causes generation of reactive oxygen species (ROS) that trigger renal tubular injury. We found that rapamycin, an inhibitor of mTORC1, attenuated Cd-induced apoptosis in renal tubular cells. Knockdown of Raptor, a positive regulator of mTORC1, also had the similar effect. However, rapamycin did not alter generation of ROS, suggesting that mTORC1 is a target downstream of ROS. Indeed, ROS caused activation of mTORC1, which contributed to induction of a selective branch of the unfolded protein response (UPR); *i.e.*, the IRE1 pathway. Although Cd triggered three major UPR pathways, activation of mTORC1 by Cd did not contribute to induction of the PERK–eIF2α and ATF6 pathways. Consistently, knockdown of Raptor caused suppression of JNK without affecting the PERK–eIF2α pathway in Cd-exposed cells. Knockdown of TSC2, a negative regulator of mTORC1, caused activation of mTORC1 and enhanced Cd induction of the IRE1–JNK pathway and apoptosis without affecting other UPR branches. Inhibition of IRE1α kinase led to suppression of JNK activity and apoptosis in Cd-treated cells. Dominant-negative inhibition of JNK also suppressed Cd-induced apoptosis. In contrast, inhibition of IRE1α endoribonuclease activity or downstream XBP1 modestly enhanced Cd-induced apoptosis. *In vivo*, administration with rapamycin suppressed activation of mTORC1 and JNK, but not eIF2α, in the kidney of Cd-treated mice. It was correlated with attenuation of tubular injury and apoptotic cell death in the tubules. These results elucidate dual regulation of Cd-induced renal injury by mTORC1 through selective induction of IRE1 signaling.

## Introduction

Cadmium (Cd) is one of highly toxic metals and accumulates in a variety of organs, especially in the kidney, through cigarette smoking and drinking of contaminated water [1], [2]. Cd exposure is nephrotoxic and causes renal tubular damage, the main target of the Cd-related toxicity. In the kidney, accumulation of Cd occurs mainly in the proximal tubules, and other nephron segments are affected only at later stages of intoxication [3].

It is known that Cd toxicity is mediated by oxidative stress. A line of evidence showed that Cd alters antioxidant defense mechanisms and increases generation of reactive oxygen species (ROS) including superoxide anion and hydrogen peroxide [4]–[7]. ROS have the potential to modify proteins, lipids and DNA, leading to apoptotic cell death via several stress pathways including the endoplasmic reticulum (ER) stress response [8]–[11].

ER stress is caused by accumulation of unfolded or misfolded proteins in the ER and implicated in the induction of apoptosis under a wide range of pathological situations [12], [13]. ER stress induces the unfolded protein response (UPR) that inhibits or facilitates apoptosis [14].The UPR comprises three major signaling pathways initiated by inositol-requiring enzyme 1 (IRE1), RNA-dependent protein kinase-like ER kinase (PERK) and activating transcription factor 6 (ATF6) [15]. IRE1 catalyzes removal of a 26-nucleotide intron from the X-box binding protein 1 (*XBP1*) mRNA. This molecular event depends on the endoribonuclease (RNase) domain of IRE1 and leads to production of active XBP1 and consequent activation of ER-associated protein degradation (ERAD). Activation of PERK leads to phosphorylation of eukaryotic translation initiation factor 2α (eIF2α) and suppresses global protein translation. In response to ER stress, ATF6 is transported to the Golgi, cleaved by S1P and S2P proteases and relocates to the nucleus, leading to induction of ER chaperones such as 78 kDa glucose-regulated protein (GRP78) and GRP94. These branches of the UPR contribute to alleviation of ER stress [15], [16]. However, distinct pro-apoptotic UPR is also induced during ER stress. It includes; 1) activation of caspase-12 through a Ca^2+^-dependent pathway, 2) activation of apoptosis signal-regulating kinase 1 (ASK1) and c-Jun N-terminal kinase (JNK) via interaction of IRE1α with tumor necrosis factor receptor-associated factor 2 (TRAF2), and induction of pro-apoptotic CCAAT/enhancer-binding protein-homologous protein (CHOP) by PERK–eIF2α signaling. These UPR pathways contribute to apoptosis during ER stress [15], [16].

Mammalian target of rapamycin complex 1 (mTORC1) is composed of mTOR, regulatory associated protein of mTOR (raptor) and mLST8, which is sensitive to a macrolide antibiotic agent, rapamycin. mTORC1 is one of key regulators for cell growth and metabolism through direct phosphorylation of ribosomal p70S6 kinase (p70S6K) and eIF4E-binding protein [17], [18]. A recent report indicated that Cd has the potential to activate mTORC1, which might be involved in apoptosis of neuronal cells [19]. However, mechanisms underlying mTORC1-induced apoptosis are largely unknown. In the present investigation, we aimed at examining how ROS and mTORC1 regulate three major branches of the UPR in Cd-exposed renal tubular cells. Our current results demonstrate dual regulation of Cd-induced apoptosis by mTORC1 through selective induction of IRE1 signaling *in vitro* and *in vivo*.

## Materials and Methods

### Reagents

Rapamycin, cadmium chloride, N-acetylcysteine (NAC), menadione and 2,3-dimethoxy-1,4-naphthoquinone (DMNQ) were purchased from Sigma-Aldrich Japan (Tokyo, Japan).

### Cells and Stable Transfectants

The rat renal tubular epithelial cell line NRK-52E was purchased from American Type Culture Collection (Manassas, VA). Cells were maintained in Dulbecco's modified Eagle's medium/Ham's F-12 (Gibco-BRL, Gaithersburg, MD) supplemented with 5 % fetal bovine serum (FBS). All experiments were performed in the presence of 1 % FBS.

### Transient Transfection

Using GeneJuice Transfection Reagent (Novagen, Madison, WI), cells were transiently transfected with XBP1(S), a spliced form of XBP1 (provided by Dr. Akihiro Tomida, Japanese Foundation of Cancer Research, Tokyo, Japan) [20]; XBP1-DN, a dominant-negative mutant of XBP1 (provided by Dr. Laurie H. Glimcher, Harvard Medical School, MA) [21]; IRE1αK599A, a kinase-defective mutant of IRE1α (provided by Dr. Masayuki Miura, University of Tokyo, Tokyo, Japan) [22]; IRE1αΔRNase, RNase domain-deleted mutant of IRE1α (provided by Dr. Kazunori Imaizumi, University of Hiroshima, Hiroshima, Japan) [23]; JNK-DN, a dominant-negative mutant of JNK (provided by Dr. Roger J. Davis, University of Massachusetts Medical Center Worcester, MA) [24]; or siRNAs. siRNA for tuberous sclerosis complex 2 (TSC2) and Raptor were purchased from TAKARA (Shiga, Japan). The nucleotide sequences are: siTSC2, 5′-GGCCCUCACAGACAAUGGA-3′; and siRaptor, 5′-GCCUGAGUCUGUGAAUGUA-3′ [25]. 5′-GCUGCAAUCGAUUGAUAGC-3′ was used as a control siRNA.

### Western Blot Analysis

Western blot analysis was performed as described previously [26]. Anti-caspase-3, anti-phospho-PERK (Thr980), anti-phospho-eIF2α (Ser51), anti-phospho-JNK (Thr183/Tyr185), anti-JNK, anti-phospho-p70S6K (Thr389), anti-p70S6K, anti-phospho-p38 MAPK (Thr180/Thr182), anti-p38 MAPK and anti-TSC2 antibodies were purchased from Cell Signaling Technology (Beverly, MA). Anti-PERK, anti-XBP1, anti-ATF4 (CREB-2), anti-eIF2α and anti-GRP78 antibodies were purchased from Santa Cruz Biotechnology (Santa Cruz, CA). ATF6 was detected by anti-ATF6 antibody (IMGENEX, San Diego, CA). As loading controls, levels of β-actin and lamin B1 were evaluated using anti-β-actin (Sigma-Aldrich Japan) and anti-lamin B1 antibodies (Invitrogen, Carlsbad, CA). Densitometric analysis was performed using ImageJ Software (National Institutes of Health, Bethesda, MD). Nuclear protein extraction was performed using the ProteoExtract Subcellular Proteome Extraction Kit (Calbiochem, San Diego, CA) according to the manufacturer's protocol.

### RT-PCR

Reverse transcription was performed using PrimeScript 1st Strand cDNA Synthesis Kit (Takara, Ootsu, Japan). RT-PCR of *XBP1* mRNAs was performed as described previously [26].

### Assessment of Cell Death

After exposure to Cd, morphologic examination was performed by phase-contrast microscopy. The number of viable cells (both attached cells and floating cells) was estimated by trypan blue exclusion. Cleavage of procaspase-3 was used as another indicator for apoptosis.

### Detection of ROS

Generation of ROS was detected using Total ROS/Superoxide Detection Kit (Enzo Life Sciences, Farmingdale, NY). Cells were loaded with ROS-responsive fluorescence probe for 1 hr. After washing with phosphate-buffered saline (PBS), cells were exposed to Cd for 6 hr and subjected to fluorescence microscopy.

### Animal Experiment

C57BL/6 mice (20–25 g body weight; 20 male mice) were intraperitoneally injected (i.p.) with PBS or rapamycin (1.5 mg/kg) on day 1, 2, 3 and 4. On day 2, mice were administrated twice with Cd (10 mg/kg, i.p.) at an interval of 12 hr. After 3 days (day 5), kidneys were removed and processed for tissue sectioning for histopathological analysis and terminal deoxynucleotidyl transferase-mediated dUTP-biotin nick end labeling (TUNEL) assay, as described below. Renal cortex and liver were also used for Western blot analysis. All animal experiments were approved by the Animal Experiment Committee of the University of Yamanashi. All efforts were made to minimize suffering.

### Histopathological Analysis and TUNEL Assay

Kidneys were fixed in 4 % phosphate-buffered paraformaldehyde overnight at 4°C and embedded in paraffin. Tissue sections were stained with hematoxylin and eosin (HE). TUNEL assay was performed as described previously [26]. TUNEL stained sections were mounted in VECTASHIELD mounting medium (Vector Laboratories, Burlingame, CA). The number of TUNEL-positive cells per field was counted and compared the data in different groups.

### Statistical Analysis

Assessment of cell death was performed in quadruplicate. Data were presented as means ± SE. Statistical analysis was performed using non-parametric Mann-Whitney *U* test to compare data in different groups. *P* value <0.05 was considered to indicate a statistically significant difference.

## Results

### Involvement of mTORC1 in Cd-triggered Apoptosis

**Figure 1:**
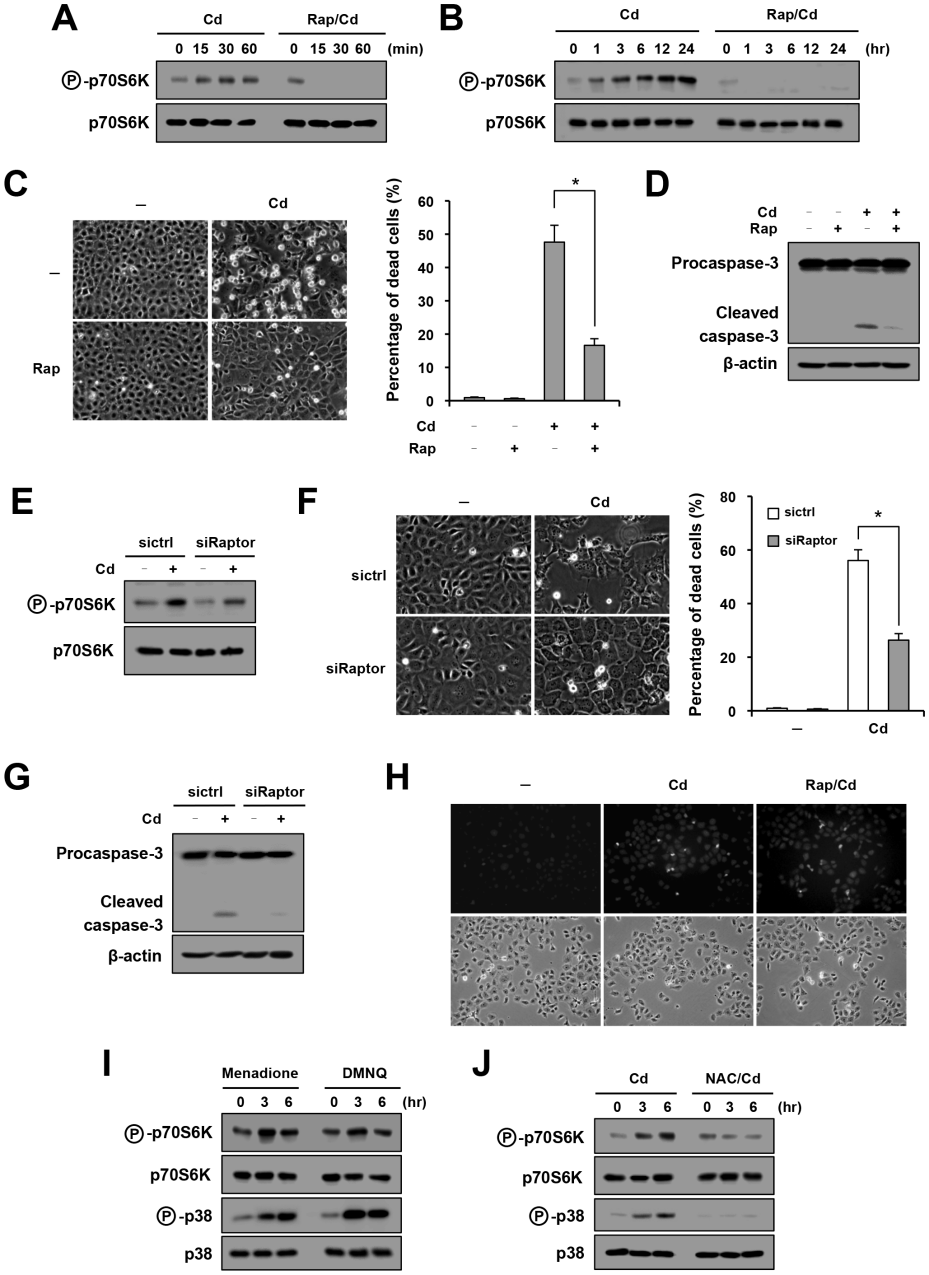
Involvement of mTORC1 in Cd-triggered apoptosis. (A, B) NRK-52E cells were exposed to cadmium (Cd; 10 µM) in the absence or presence of rapamycin (Rap; 100 nM) for indicated time periods and subjected to Western blot analysis of phosphorylated p70S6K. The level of total p70S6K protein is shown at the bottom as a loading control. (C) Cells were treated with Cd in the absence or presence of rapamycin for 72 hr and subjected to phase-contrast microscopy (left) and trypan blue analysis for quantitative assessment of cellular death (right). Assays were performed in quadruplicate, and data are shown as means ± SE. An asterisk indicates a statistically significant differences (*p*<0.05). (D) Cells were treated with indicated agents for 48 hr, and Western blot analysis was performed to analyze cleavage of procaspase-3. The level of β-actin is shown at the bottom as a loading control. (E–G) Cells were transfected with siRaptor (20 nM) or control siRNA (sictrl; 20 nM), treated with Cd and subjected to Western blot analysis of phosphorylated p70S6K (E), assessment of cell death (F) and analysis of procaspase-3 cleavage (G). (H) Cells were loaded with ROS-responsive fluorescence probe for 1 hr, treated with Cd and rapamycin for 6 hr and subjected to fluorescence microscopy. Light microscopic image is shown at the bottom. (I, J) Cells were treated with indicated reagents [menadione (10 µM), DMNQ (5 µM), NAC (1 mM; pretreatment for 1 hr), Cd (10 µM)] for indicated time periods and subjected to Western blot analysis of phosphorylated p70S6K and p38 MAPK.

A previous report indicated activation of mTORC1 by Cd in neuronal cell lines [19]. We first examined activation of mTORC1 after exposure to Cd in NRK-52E renal tubular cells. Phosphorylation of p70S6K was used as an indicator for mTORC1 activation. As shown in Figure 1A, activity of mTORC1 was rapidly up-regulated following exposure to Cd, and it was completely abrogated by the treatment with rapamycin. The activation progressed in a time-dependent manner for at least 24 hr (Figure 1B).

We next examined an effect of rapamycin on Cd-triggered cellular death. Microscopic analysis showed that rapamycin significantly attenuated Cd-induced cell injury (Figure 1C). It was associated with inhibition of caspase-3 activation (Figure 1D), suggesting that rapamycin suppressed Cd-induced apoptosis. Raptor is an essential component of mTORC1, and knockdown of Raptor by siRNA attenuated Cd-induced activation of mTORC1 (Figure 1E). The down-regulation of mTORC1 by Raptor siRNA significantly suppressed cellular death and activation of caspase-3 caused by Cd (Figures 1F and 1G). These results suggest that mTORC1 plays a crucial role in Cd-triggered apoptosis of renal tubular cells.

It is known that Cd causes apoptosis through generation of ROS [27]. We examined whether or not rapamycin alters Cd-triggered generation of ROS. For this purpose, cells were loaded with a ROS-responsive fluorescence probe and exposed to Cd. As shown in Figure 1H, generation of ROS was induced by exposure to Cd, whereas it was unaffected by the treatment of rapamycin. This result suggested that mTORC1 is involved in Cd-induced apoptosis downstream of oxidative stress. To examine this possibility, we tested the potential of ROS to activate mTORC1. Cells were treated with ROS generators menadione and DMNQ [28], [29], and phosphorylation of p70S6K was evaluated. Western blot analysis showed that menadione or DMNQ induced phosphorylation of p70S6K and p38 mitogen-activated protein kinase (p38 MAPK), the latter of which is an endogenous marker for oxidative stress [30] (Figure 1I). Of note, phosphorylation of p70S6K and p38 MAPK by Cd was completely abolished by the treatment with antioxidant NAC (Figure 1J).

### Selective Induction of the IRE1 Pathway by mTORC1

**Figure 2:**
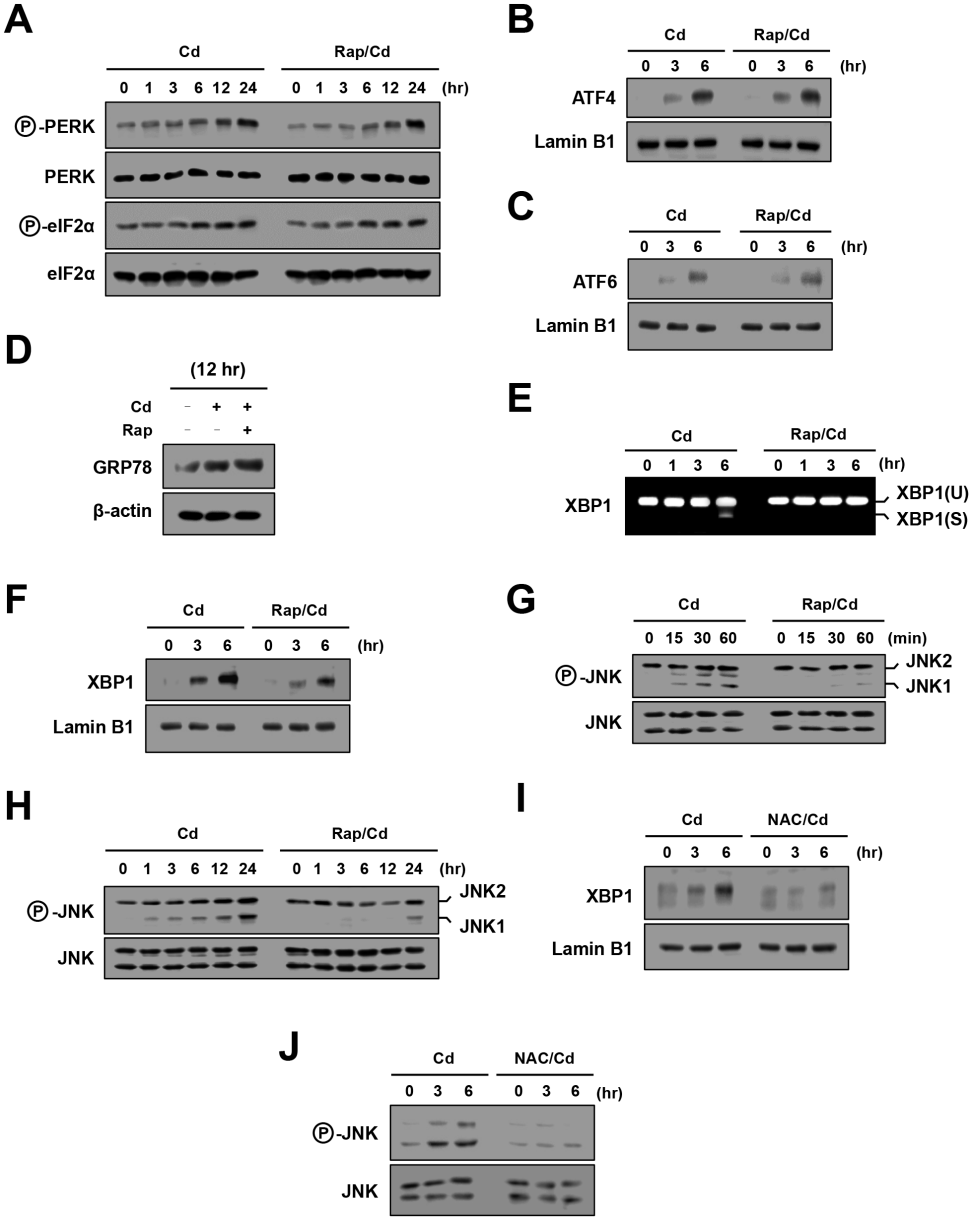
Selective induction of the IRE1 pathway by mTORC1 during Cd exposure. Cells were treated with Cd together with or without rapamycin for indicated time periods and subjected to RT-PCR analysis of *XBP1* mRNA (E) and Western blot analysis of phosphorylated PERK and phosphorylated eIF2α (A), nuclear ATF4 (B), nuclear ATF6 (C), total GRP78 (D), nuclear XBP1 (F) and phosphorylated JNK (G, H). As loading controls, protein levels of PERK, eIF2α, lamin B1 and JNK are shown. XBP1(U), unspliced form of *XBP1*; XBP1(S), spliced form of *XBP1*. (I, J) Cells were pretreated with NAC for 1 hr, treated with Cd for 6 hr and subjected to Western blot analysis of nuclear XBP1 (I) and phosphorylated JNK (J).

ROS have the potential to induce ER stress [31]. To identify UPR pathways responsible for the mTORC1-mediated pro-apoptotic effect of Cd, we first examined a role of the PERK–eIF2α pathway. Exposure of cells to Cd induced phosphorylation of PERK and eIF2α. However, inhibition of mTORC1 by rapamycin did not affect this induction (Figure 2A). Consistent with this result, Cd-triggered nuclear translocation of ATF4, a signaling event downstream of eIF2α, was not affected by rapamycin (Figure 2B). These results suggested lack of involvement of the PERK–eIF2α pathway in the mTORC1-mediated pro-apoptotic effect of Cd.

We next examined a role of the ATF6 pathway. Exposure of cells to Cd caused nuclear translocation of ATF6. However, this process was not affected by inhibition of mTORC1 by rapamycin (Figure 2C). Consistent with this result, Cd-triggered induction of GRP78, an event downstream of ATF6, was not altered by rapamycin (Figure 2D). These results suggested that the ATF6 pathway was not involved in the mTORC1-mediated pro-apoptotic effect of Cd.

We investigated a role of the IRE1 pathway in the pro-apoptotic effect of mTORC1 in Cd-exposed cells. For this purpose, splicing of *XBP1* mRNA and nuclear accumulation of XBP1 were used as indicators for IRE1α RNase activity. As shown in Figures 2E and 2F, both splicing of *XBP1* and nuclear accumulation of XBP1 were induced by Cd. In contrast to other UPR branches, the induction and activation of XBP1 via IRE1α were inhibited by the treatment with rapamycin. We further examined an effect of rapamycin on IRE1α kinase activity. The kinase domain of IRE1 activates JNK via interaction with TRAF2 and ASK1, contributing to ER stress-induced apoptosis [32]. Western blot analysis showed that phosphorylation of JNK1 and JNK2, especially JNK1, was rapidly induced by Cd (Figure 2G). This activation was progressed for at least 24 hr (Figure 2H). When mTORC1 was inhibited by rapamycin, phosphorylation of JNK was markedly diminished (Figures 2G and 2H). Of note, scavenging of ROS by NAC also attenuated nuclear translocation of XBP1 (Figure 2I) and phosphorylation of JNK (Figure 2J) in Cd-exposed cells.

**Figure 3:**
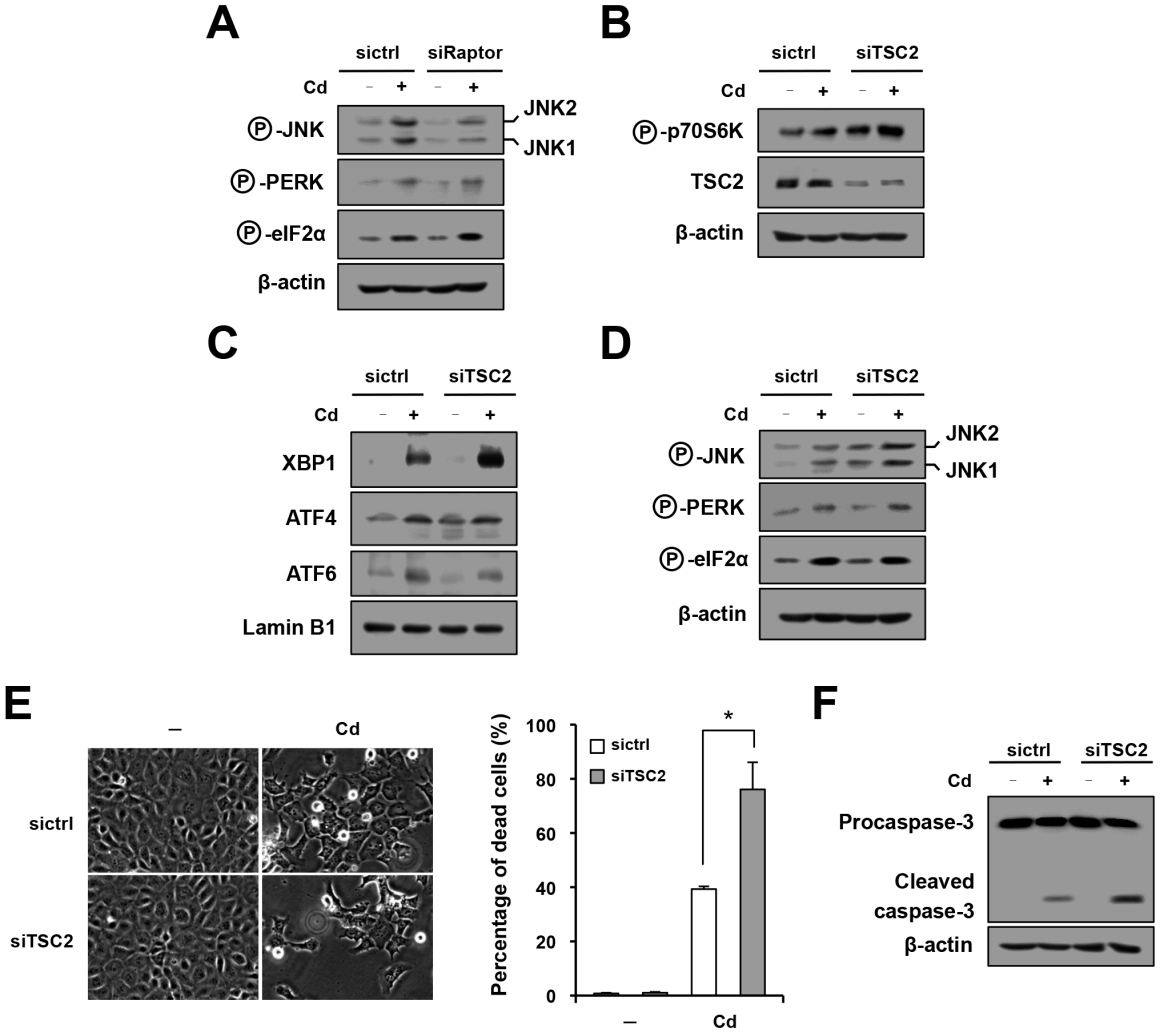
Selective induction of the IRE1 pathway and apoptosis by mTORC1 activated by Cd. (A) Cells were transfected with sictrl and siRaptor (A) or siTSC2 (B–D), treated with Cd for 6 hr and subjected to Western blot analysis of the molecules indicated on the left. In (C), nuclear proteins were used for analysis. (E, F) The sictrl- and siTSC2-transfected cells were treated with Cd and subjected to phase-contrast microscopy (E, left), trypan blue analysis (E, right) and Western blot analysis of caspase-3 (F).

To confirm selective involvement of the IRE1 pathway in Cd-triggered, mTORC1-related apoptosis, cells were transfected with siRaptor, stimulated with Cd and subjected to Western blot analysis. Consistent with the results using rapamycin, suppression of mTORC1 by knockdown of Raptor led to blunted activation of JNK by Cd without affecting the PERK–eIF2α pathway (Figure 3A). To further confirm this result, we also employed siRNA to knockdown TSC2, a negative regulator for mTORC1 [33]. Down-regulation of TSC2 and up-regulation of mTORC1 activity by siTSC2 were confirmed by Western blot analysis (Figure 3B). In the cells transfected with siTSC2, Cd-induced nuclear accumulation of XBP1 was enhanced, whereas nuclear translocation of ATF4 and ATF6 was unaffected (Figure 3C). Similarly, phosphorylation of JNK1 and JNK2 in response to Cd was also reinforced in TSC2-knockdown cells, whereas phosphorylation of PERK and eIF2α was unaffected (Figure 3D). The selective reinforcement of the mTORC1–IRE1 pathway by siTSC2 was associated with accelerated cell death in Cd-exposed cells. As shown in Figure 3E, siTSC2 significantly enhanced percentages of dead cells following exposure to Cd. Western blot analysis also showed that cleavage of procaspase-3 was enhanced in TSC2-knockdown cells (Figure 3F). These data further support the idea that, in Cd-exposed cells, activated mTORC1 selectively induces the IRE1 pathway and thereby causes apoptosis. It is consistent with our previous finding that mTORC1 is required for activation of IRE1α in ER stress-exposed cells [26].

### Dual Regulation of Cd-induced Apoptosis by IRE1

**Figure 4:**
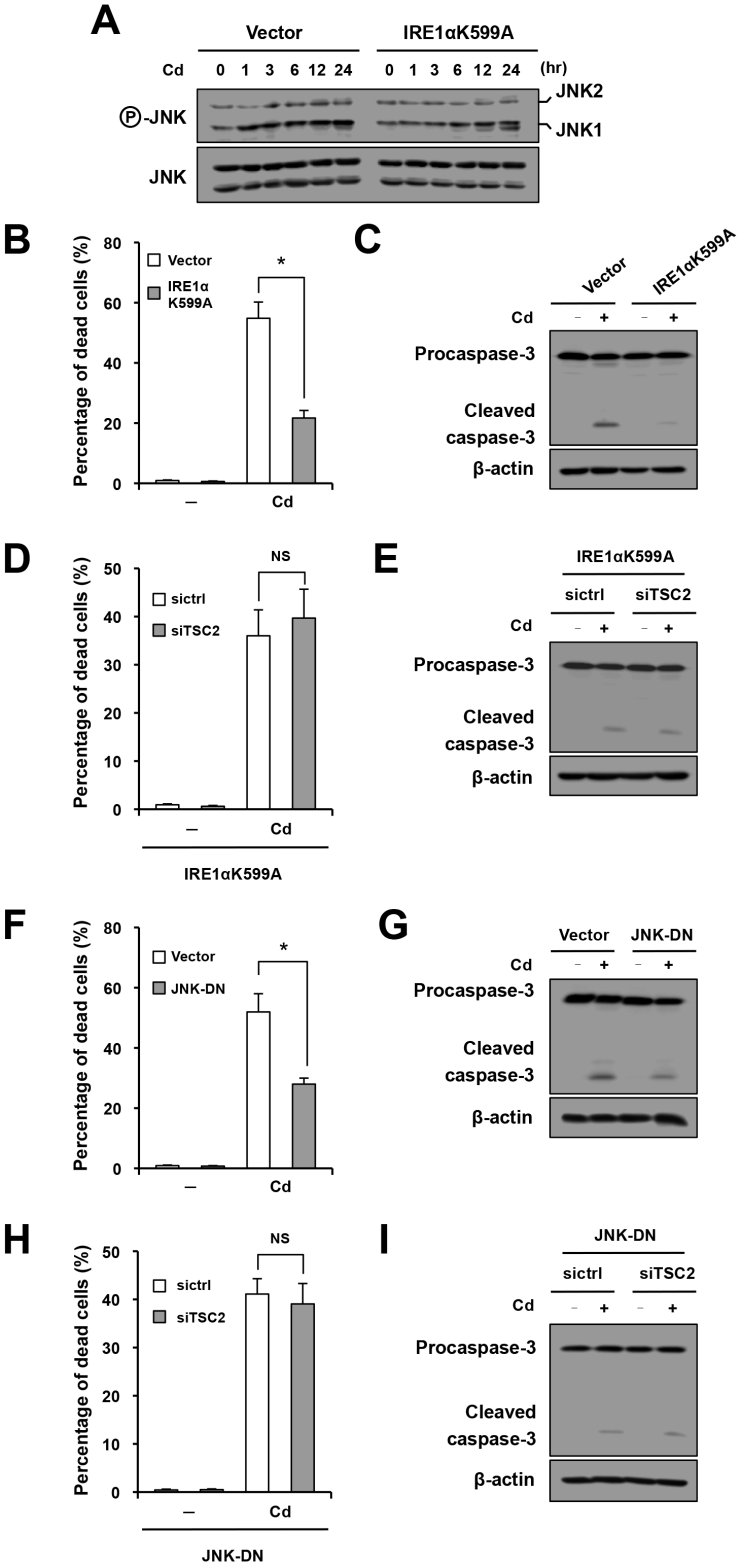
Pro-apoptotic role of the IRE1–JNK pathway in Cd-triggered, mTORC1-mediated apoptosis. (A–C, F, G) Cells were transfected with empty vector (Vector) and IRE1αK599A (A–C) or dominant-negative mutant of JNK1 (JNK-DN) (F, G), treated with Cd for indicated time periods (A) or 60 – 72 hr (B, C, F, G) and subjected to Western blot analysis of phosphorylated JNK (A), trypan blue analysis (B, F) and assessment of procaspase-3 cleavage (C, G). (D, E, H, I) Cells were co-transfected with IRE1αK599A and siTSC2 (D, E) or JNK-DN and siTSC2 (H, I), treated with Cd for 60 – 72 hr, and subjected to trypan blue analysis (D, H) and Western blot analysis of caspase-3 (E, I). NS, not statistically significant.

The IRE1 pathway may modulate apoptotic processes positively or negatively, depending on different cellular contexts. In some situations, the IRE1–JNK pathway is considered to be pro-apoptotic, whereas the IRE1–XBP1 pathway may be anti-apoptotic via induction of ER chaperones and ERAD factors [14], [16]. However, in other situations, XBP1 may function as a pro-apoptotic molecule [34], and JNK could contribute to cell survival [35]. To determine exact contribution of IRE1 signaling to the control of Cd-triggered apoptosis, we first focused on the function of the IRE1α kinase domain. For this purpose, cells were transfected with a kinase-defective mutant of IRE1α (IRE1αK599A). Effective suppression of IRE1α kinase activity was confirmed by Western blot analysis using JNK phosphorylation as an indicator (Figure 4A). As shown in Figure 4B, dominant-negative inhibition of IRE1α kinase resulted in suppression of Cd-triggered cellular death. It was associated with attenuation of caspase-3 activation (Figure 4C). Under the suppression of IRE1α kinase activity, the pro-apoptotic effect of siTSC2 was abolished (Figures 4D and 4E), indicating that IRE1α kinase activity is required for Cd-triggered, mTORC1-related apoptosis. To further confirm this result, cells were transfected with a dominant-negative mutant of JNK, and the apoptotic response to Cd was retested. As shown in Figures 4F and 4G, suppression of JNK led to significant inhibition of cellular death and cleavage of procaspase-3 in Cd-exposed cells. Of note, under the suppression of JNK, the pro-apoptotic effect of siTSC2 was abolished (Figures 4H and 4I). Taken together, these results suggest that the IRE1–JNK pathway is required for Cd-triggered, mTORC1-related apoptosis.

**Figure 5:**
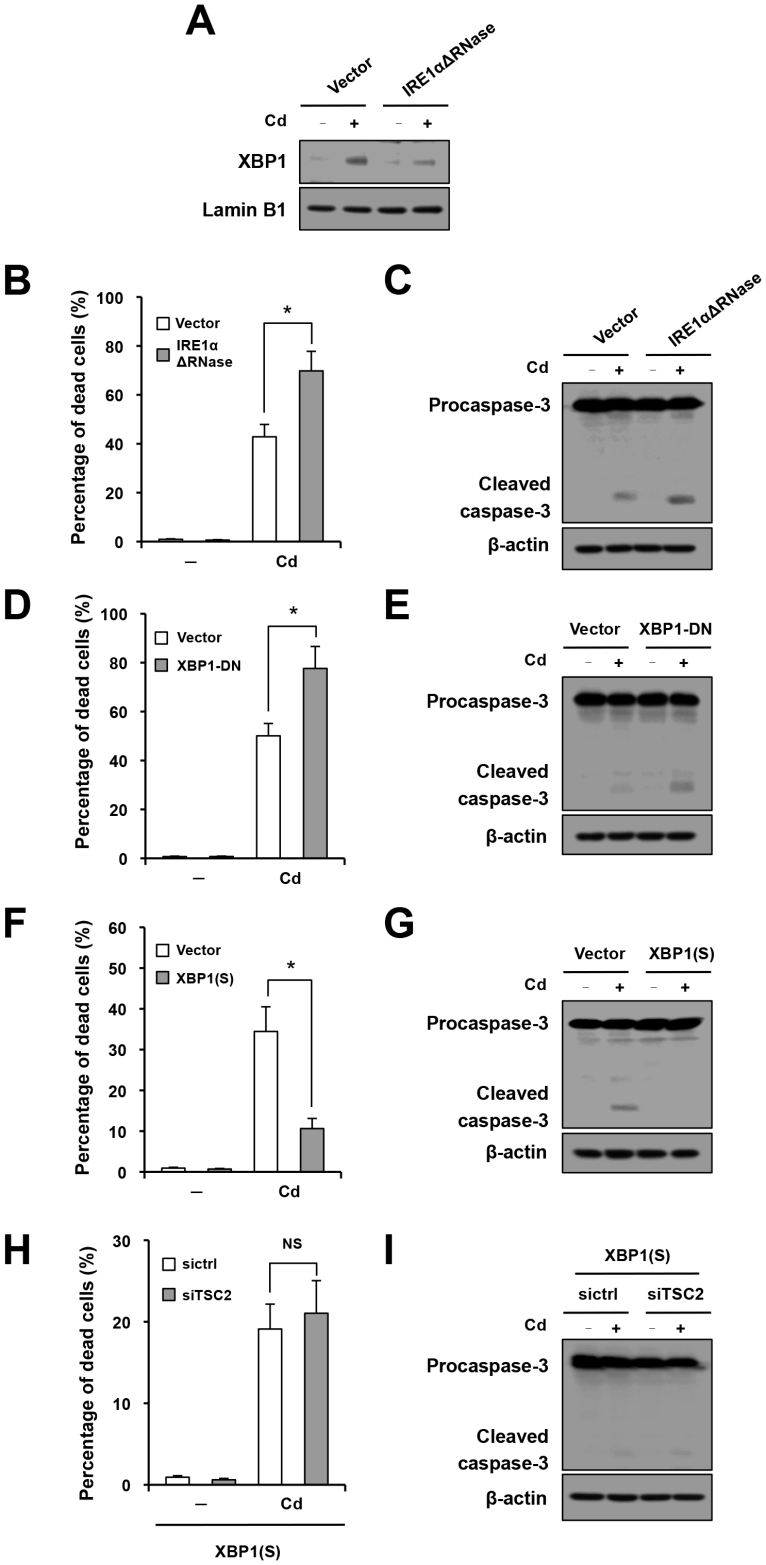
Anti-apoptotic role of the IRE1–XBP1 pathway that counteracts the pro-apoptotic IRE1–JNK. (A–G) Cells were transfected with empty vector and IRE1αΔRNase (A–C), dominant-negative mutant of XBP1 (XBP1-DN) (D, E) or XBP1(S) (F, G), treated with Cd for 6 hr (A) or 60 hr (B–G) and subjected to Western blot analysis of nuclear XBP1 (A), trypan blue analysis (B, D, F) and assessment of procaspase-3 cleavage (C, E, G). (H, I) Cells were co-transfected with XBP1(S) and siTSC2, treated with Cd for 60 hr, and subjected to trypan blue analysis (H) and Western blot analysis of caspase-3 (I).

We next focused on the function of the IRE1α RNase domain. For this purpose, cells were transfected with an RNase-deleted mutant of IRE1α (IRE1αΔRNase). Effective suppression of IRE1α RNase activity was confirmed by Western blot analysis using nuclear XBP1 as an indicator (Figure 5A). As shown in Figure 5B, dominant-negative inhibition of IRE1α RNase resulted in enhancement of Cd-triggered cellular death. It was associated with increased caspase-3 activation (Figure 5C). To further confirm this result, cells were transfected with a dominant-negative mutant of XBP1, and the apoptotic response to Cd was retested. As shown in Figures 5D and 5E, suppression of XBP1 led to significant increases in cellular death and cleavage of procaspase-3 in Cd-exposed cells. Furthermore, apoptotic cell death in Cd-exposed cells was substantially inhibited by transfection with *XBP1(S)*, a gene encoding the spliced form of XBP1 (Figures 5F and 5G). Of note, under overexpression of *XBP1(S)*, the pro-apoptotic effect of siTSC2 was abolished (Figures 5H and 5I). These results suggest that, in contrast to the IRE1–JNK pathway, the IRE1–XBP1 pathway counteracts against Cd-triggered, mTORC1-related apoptosis.

### *In Vivo* Suppression of Cd-triggered Apoptosis in Renal Tubules by Blockade of mTORC1

**Figure 6:**
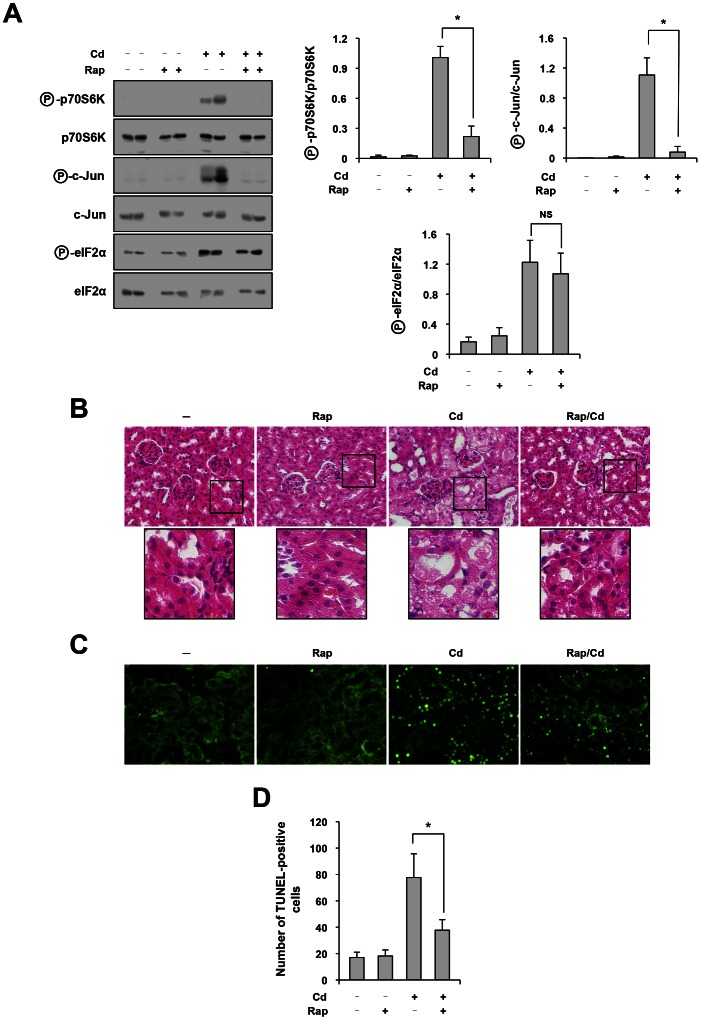
*In vivo* suppression of Cd-triggered apoptosis in renal tubules by blockade of mTORC1. Mice (5 mice/group) were injected with PBS or rapamycin (1.5 mg/kg) intraperitoneally on day 1, 2, 3 and 4. On day 2, mice were administered twice with Cd (10 mg/kg) at an interval of 12 hr. After 3 days (day 5), renal cortex were subjected to Western blot analysis of molecules indicated on the left (A), histopathological analysis (HE staining) (B) and TUNEL assay (C). In (A), quantitative analysis of individual bands is shown in the right graphs. In (D), the number of TUNEL-positive cells per field was evaluated quantitatively. Data are expressed as means ± SE, and an asterisk indicates a statistically significant difference (*p*<0.05).

We examined whether *in vivo* inhibition of mTORC1 can block Cd-triggered, IRE1–JNK-mediated pro-apoptotic pathway in renal tubules. For this purpose, mice were injected with PBS or rapamycin intraperitoneally on days 1, 2, 3 and 4. On day 2, mice were exposed to Cd. After 3 days (day 5), kidneys were removed and processed for Western blot analysis and tissue sectioning for histopathological analysis and TUNEL assay. As shown in Figure 6A, activation of mTORC1 was induced in the kidneys after exposure to Cd, which was abolished by administration with rapamycin (top row). Consistent with our *in vitro* findings, inhibition of mTORC1 resulted in suppression of JNK activation indicated by the level of phosphorylated c-Jun (Figure 6A, third row). In contrast, phosphorylation of eIF2α by Cd was not affected by rapamycin (Figure 6A, fifth row). It suggests that, like in cultured tubular cells, mTORC1 is not involved in the activation of the PERK–eIF2α pathway by Cd. Of note, the same results were also observed in the liver (data not shown). In consistent with this finding, histopathological analysis showed that injury of the renal tubules by Cd was markedly attenuated by administration with rapamycin (Figure 6B). Furthermore, TUNEL-positive apoptotic cells increased in Cd-exposed tubules, which was significantly attenuated by rapamycin (Figure 6C). The number of TUNEL-positive cells significantly decreased by the treatment with rapamycin from 78±21 cells/field to 38±8 cells/field (Figure 6D). These results provide *in vivo* evidence supporting our conclusion that mTORC1 causes apoptosis of renal tubular cells through induction of the selective UPR, *i.e*., the IRE1–JNK pathway.

## Discussion

**Figure 7:**
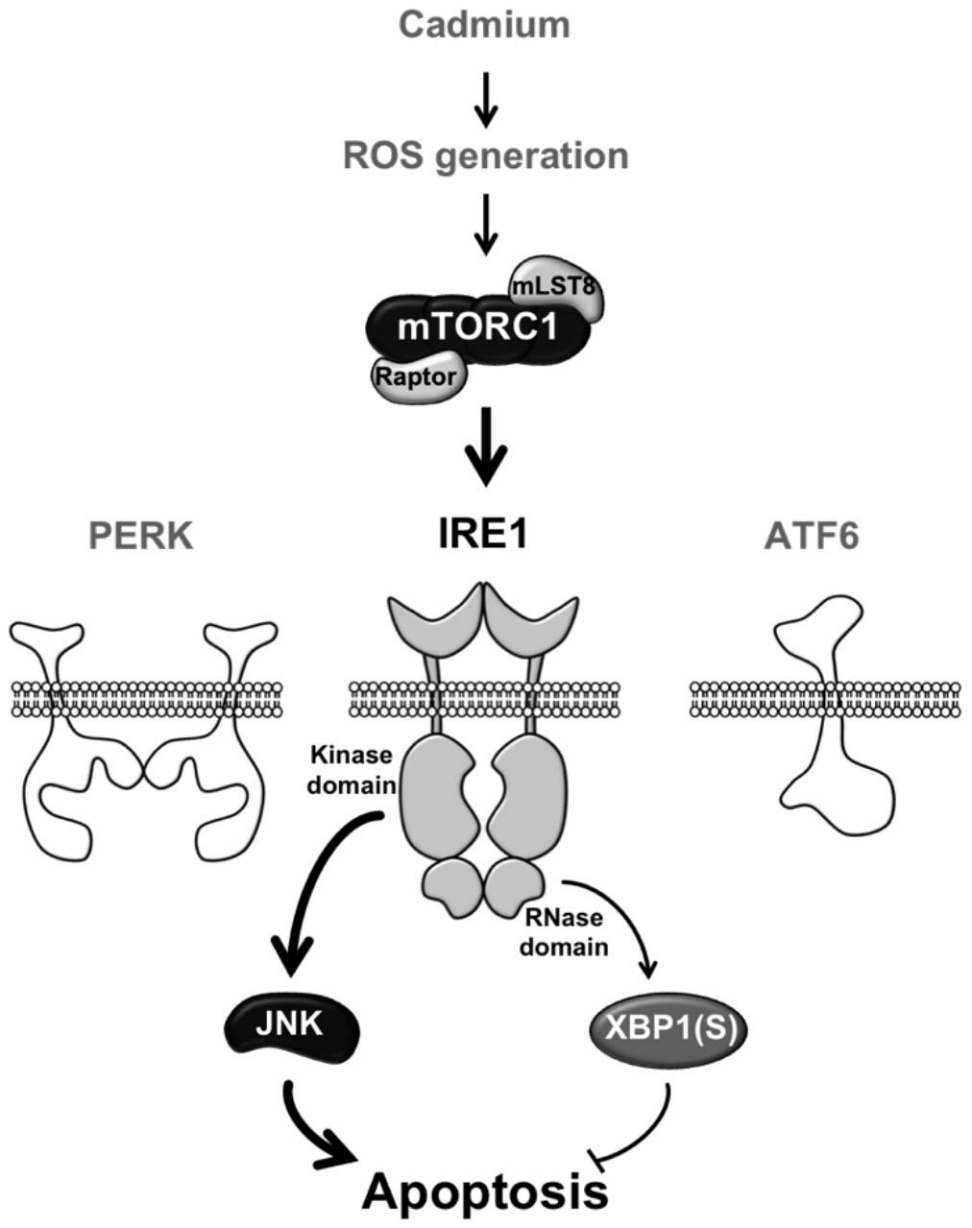
Dual regulation of Cd-induced apoptosis by mTORC1 through selective induction of the IRE1 pathways. Cd induces activation of mTORC1 through generation of ROS. mTORC1 subsequently activates IRE1 without affecting PERK and ATF6, although mTORC1-independent mechanisms may also be involved in Cd/ROS-induced IRE1 activation. Activation of the IRE1 at the kinase domain leads to phosphorylation of JNK, leading to apoptotic cell death. On the other hand, activation of the IRE1 at the RNase domain causes generation of active XBP1. This IRE1–XBP1 pathway modestly counteracts the pro-apoptotic action of IRE1–JNK signaling.

In the present report, we disclosed a crucial role of the mTORC1–IRE1 pathway in the regulation of Cd-triggered apoptosis *in vitro* and *in vivo*. We found; 1) under the exposure to Cd, mTORC1 is activated through generation of ROS, 2) activation of mTORC1 selectively induces the IRE1 pathway, but not the PERK and ATF6 pathways, and 3) the IRE1–JNK pathway is the major pro-apoptotic pathway in Cd-triggered, mTORC1-mediated apoptosis, which is partially counteracted by the IRE1–XBP1 anti-apoptotic pathway. The outline of our current findings is illustrated in Figure 7.

Generation of ROS plays a crucial role in Cd-induced apoptosis [27]. ROS may directly induce cellular death through oxidative damage of proteins, lipids and nucleic acids [10]. However, recent investigation indicated more complicated mechanisms underlying Cd-induced apoptosis. For example, some investigators suggested involvement of mTORC1, and others indicated a role of ER stress in some cell types [19], [31], [36]. Currently, linkage among oxidative stress, ER stress and mTORC1 has not been fully elucidated. A previous report showed that ROS activated mTORC1 via activation of phosphoinositide 3 kinase, a positive regulator of mTORC1, and via inhibition of AMP-activated protein kinase, a negative regulator of mTORC1 [37]. However, it is still unclear how mTORC1 contributes to Cd-triggered apoptosis. In the present report, we showed that ROS-mediated activation of mTORC1 caused selective induction of the pro-apoptotic branch of the UPR, the IRE1–JNK pathway, leading to apoptosis.

In this report, we showed that mTORC1 has the potential to up-regulate both RNase and kinase activities of IRE1α without affecting other UPR branches. It means that mTORC1 triggers activation of IRE1α without induction of ER stress. If so, how does mTORC1 activate IRE1α? Some previous report suggested that action of IRE1 (as RNase and kinase) requires binding of adaptor proteins such as Bcl-2 family proteins Bax and Bak to the C-terminal of IRE1α [38]. Lam *et al.* reported that the mTORC1 pathway contributes to up-regulation of BH3-only proteins (*e.g.,* Bim and Puma) that increase the level of Bax and Bak in the ER. In the ER, these molecules are up-regulated by BH3-only proteins [39]–[41]. mTORC1 may cause activation of IRE1α through up-regulation of these adaptor proteins.

Individual UPR pathways possess both pro-apoptotic and anti-apoptotic properties. Lin *et al.* reported that, under persistent ER stress, activation of IRE1α was only transient and attenuated in the later phase in HEK293 cells. When IRE1α activity was sustained artificially, cell survival was rather enhanced, suggesting the pro-survival role of the IRE1 pathway [42]. In the current report, however, we showed that activation of mTORC1 by Cd caused induction of the pro-apoptotic IRE1 pathway in NRK-52E cells. We also found that the pro-apoptotic process is a little complicated. The IRE1α kinase domain contributes to apoptosis through activation of JNK, whereas the IRE1α RNase domain counteracts modestly against apoptosis via induction of XBP1. The anti-apoptotic effect of XBP1 may be via induction of ER chaperones and ERAD factors that attenuate ER stress [43].

Currently, it is unclear why the IRE1 pathway is anti-apoptotic in some cellular contexts, whereas it turns around to be pro-apoptotic in other situations. On possibility is that the UPR may be different depending on cell types and/or triggers. For example, in HEK293 cells exposed to relatively high concentrations of tunicamycin and thapsigargin, activation of IRE1α kinase activity was only transient [42]. In contrast, in our experimental setting, Cd caused sustained phosphorylation of JNK in NRK-52E cells. The different kinetics of JNK activation, or different balance between IRE1–XBP1 and IRE1–JNK signaling possibly results in different roles of IRE1 in regulating cellular fate.

In Figure 7, we have illustrated that mTORC1 plays a role in linking Cd/ROS signaling to IRE1 activation. However, this process may not be direct and could also be mediated by other signaling molecules. For example, a previous report showed that suppression of Akt was involved in mTORC1-induced activation of IRE1 [26]. Similarly, we cannot exclude a possibility that Cd-triggered IRE1 activation and apoptosis are mediated not only by mTORC1 but also by some mTORC1-independent mechanisms.

Cd causes ER stress and triggers three major branches of the UPR [11]. Individual UPR pathways are mainly pro-apoptotic or anti-apoptotic. For example, as demonstrated in this report, the IRE1 pathway is mainly pro-apoptotic. In contrast, the PERK pathway is mainly anti-apoptotic [11]. Towards prevention or treatment of Cd-induced renal tubular injury, selective inhibitors of the pro-apoptotic UPR may be advantageous over the use of chemical chaperones that interfere with all UPR pathways. Currently, however, little is known about agents that selectively inhibit particular pro-apoptotic branches. In the present report, we showed that *in vivo* administration with rapamycin selectively suppressed Cd-triggered activation of the IRE1–JNK signaling, but not the PERK pathway, resulting in significant attenuation of renal tubular injury. Our findings raise a possibility that rapamycin may be useful for the treatment of a wide range of ER stress-related disorders including Cd-induced tissue injury.

## References

[1] Lind Y, Engman J, Jorhem L, Glynn AW (1997) Cadmium accumulation in liver and kidney of mice exposed to the same weekly cadmium dose continuously or once a week. Food Chem Toxicol 35: 891–895. 9409629 10.1016/s0278-6915(97)00068-9

[2] Jarup L, Akesson A (2009) Current status of cadmium as an environmental health problem. Toxicol Appl Pharmaco 238: 201–208. 10.1016/j.taap.2009.04.020 19409405

[3] Bernard A (2004) Renal dysfunction induced by cadmium: biomarkers of critical effects. Bio Metals 17: 519–523. 10.1023/b:biom.0000045731.75602.b9 15688856

[4] Oh SH, Lim SC (2006) A rapid and transient ROS generation by cadmium triggers apoptosis via caspase-dependent pathway in HepG2 cells and this is inhibited through N-acetylcysteine-mediated catalase upregulation. Toxicol Appl Pharmacol 212: 212–223. 16169029 10.1016/j.taap.2005.07.018

[5] Pathak N, Khandelwal S (2006) Oxidative stress and apoptotic changes in murine splenocytes exposed to cadmium. Toxicology 220: 26–36. 16413650 10.1016/j.tox.2005.11.027

[6] Valko M, Rhodes CJ, Moncol J, Izakovic M, Mazur M (2006) Free radicals, metals and antioxidants in oxidative stress-induced cancer. Chem Biol Interact 160: 1–40. 16430879 10.1016/j.cbi.2005.12.009

[7] Ognjanović BI, Marković SD, Ethordević NZ, Trbojević IS, Stajn AS, et al (2006) Cadmium-induced lipid peroxidation and changes in antioxidant defense system in the rat testes: protective role of coenzyme Q(10) and vitamin E. Reprod Toxicol 29: 191–197. 10.1016/j.reprotox.2009.11.009 19958828

[8] Stohs SJ, Bagchi D (1995) Oxidative mechanisms in the toxicity of metal ions. Free Radic Biol Med 18: 321–336. 7744317 10.1016/0891-5849(94)00159-h

[9] Figueiredo-Pereira ME, Yakushin S, Cohen G (1998) Disruption of the intracellular sulfhydryl homeostasis by cadmium-induced oxidative stress leads to protein thiolation and ubiquitination in neuronal cells. J Biol Chem 273: 12703–12709. 9582293 10.1074/jbc.273.21.12703

[10] Valko M, Leibfritz D, Moncol J, Cronin MT, Mazur M, et al (2007) Free radicals and antioxidants in normal physiological functions and human disease. Int J Biochem Cell Biol 39: 44–84. 16978905 10.1016/j.biocel.2006.07.001

[11] Yokouchi M, Hiramatsu N, Hayakawa K, Kasai A, Takano Y, et al (2007) Atypical, bidirectional regulation of cadmium-induced apoptosis via distinct signaling of unfolded protein response. Cell Death Differ 14: 1467–1474. 17464326 10.1038/sj.cdd.4402154

[12] Lee AS (2002) The glucose-regulated proteins: stress induction and clinical applications. Trends Biochem Sci 26: 504–510. 10.1016/s0968-0004(01)01908-9 11504627

[13] Kaufman RJ (2002) Orchestrating the unfolded protein response in health and disease. J Clin Invest 110: 1389–1398. 12438434 10.1172/JCI16886 PMC151822

[14] Kim R, Emi M, Tanabe K, Murakami S (2006) Role of the unfolded protein response in cell death. Apoptosis 11: 5–13. 16374548 10.1007/s10495-005-3088-0

[15] Ron D, Walter P (2007) Signal integration in the endoplasmic reticulum unfolded protein response. Nat Rev Mol Cell Biol 8: 519–529. 17565364 10.1038/nrm2199

[16] Rutkowski DT, Kaufman RJ (2004) A trip to the ER: coping with stress. Trends Cell Biol 14: 20–28. 14729177 10.1016/j.tcb.2003.11.001

[17] Wullschleger S, Loewith R, Hall MN (2006) TOR signaling in growth and metabolism. Cell 124: 471–484. 16469695 10.1016/j.cell.2006.01.016

[18] Laplante M, Sabatini DM (2009) mTOR signaling at a glance. J Cell Sci 122: 3589–3594. 19812304 10.1242/jcs.051011 PMC2758797

[19] Chen L, Liu L, Luo Y, Huang S (2008) MAPK and mTOR pathways are involved in cadmium-induced neuronal apoptosis. J Neurochem 105: 251–261. 18021293 10.1111/j.1471-4159.2007.05133.x

[20] Park HR, Tomida A, Sato S, Tsukumo Y, Yun J, et al (2004) Effect on tumor cells of blocking survival response to glucose deprivation. J Natl Cancer Inst 96: 1300–1310. 15339968 10.1093/jnci/djh243

[21] Lee AH, Iwakoshi NN, Anderson KC, Glimcher LH (2003) Proteasome inhibitors disrupt the unfolded protein response in myeloma cells. Proc Natl Acad Sci USA 100: 9946–9951. 12902539 10.1073/pnas.1334037100 PMC187896

[22] Iwawaki T, Akai R, Kohno K, Miura M (2004) A transgenic mouse model for monitoring endoplasmic reticulum stress. Nat Med 10: 98–102. 14702639 10.1038/nm970

[23] Ogata M, Hino S, Saito A, Morikawa K, Kondo S, et al (2006) Autophagy is activated for cell survival after endoplasmic reticulum stress. Mol Cell Biol 26: 9220–9231. 17030611 10.1128/MCB.01453-06 PMC1698520

[24] Dérijard B, Hibi M, Wu IH, Barrett T, Su B, et al (1994) JNK1: a protein kinase stimulated by UV light and Ha-Ras that binds and phosphorylates the c-Jun activation domain. Cell 76: 1025–1037. 8137421 10.1016/0092-8674(94)90380-8

[25] Piao X, Kobayashi T, Wang L, Shiono M, Takagi Y, et al (2009) Regulation of folliculin (the BHD gene product) phosphorylation by Tsc2-mTOR pathway. Biochem Biophys Res Commun 389: 16–21. 19695222 10.1016/j.bbrc.2009.08.070

[26] Kato H, Nakajima S, Saito Y, Takahashi S, Katoh R, et al (2012) mTORC1 serves ER stress-triggered apoptosis via selective activation of the IRE1-JNK pathway. Cell Death Differ 19: 310–320. 21779001 10.1038/cdd.2011.98 PMC3263505

[27] Liu J, Qu W, Kadiiska MB (2009) Role of oxidative stress in cadmium toxicity and carcinogenesis. Toxicol Appl Pharmacol 238: 209–214. 19236887 10.1016/j.taap.2009.01.029 PMC4287357

[28] Sugawara M, Sugawara Y, Wen K, Giulivi C (2002) Generation of oxygen free radicals in thyroid cells and inhibition of thyroid peroxidase. Exp Biol Med 227: 141–146. 10.1177/153537020222700209 11815678

[29] Criddle DN, Gillies S, Baumgartner-Wilson HK, Jaffar M, Chinje EC, et al (2006) Menadione-induced reactive oxygen species generation via redox cycling promotes apoptosis of murine pancreatic acinar cells. J Biol Chem 281: 40485–40492. 17088248 10.1074/jbc.M607704200

[30] Yamada T, Egashira N, Bando A, Nishime Y, Tonogai Y, et al (2012) Activation of p38 MAPK by oxidative stress underlying epirubicin-induced vascular endothelial cell injury. Free Radic Biol Med 52: 1285–1293. 22330067 10.1016/j.freeradbiomed.2012.02.003

[31] Yokouchi M, Hiramatsu N, Hayakawa K, Okamura M, Du S, et al (2008) Involvement of selective reactive oxygen species upstream of proapoptotic branches of unfolded protein response. J Biol Chem 283: 4252–4260. 18086661 10.1074/jbc.M705951200

[32] Nishitoh H, Matsuzawa A, Tobiume K, Saegusa K, Takeda K, et al (2002) ASK1 is essential for endoplasmic reticulum stress-induced neuronal cell death triggered by expanded polyglutamine repeats. Genes Dev 16: 1345–1355. 12050113 10.1101/gad.992302 PMC186318

[33] Inoki K, Li Y, Zhu T, Wu J, Guan KL (2002) TSC2 is phosphorylated and inhibited by Akt and suppresses mTOR signaling. Nat Cell Biol 4: 648–657. 12172553 10.1038/ncb839

[34] Zeng L, Zampetaki A, Margariti A, Pepe AE, Alam S, et al (2009) Sustained activation of XBP1 splicing leads to endothelial apoptosis and atherosclerosis development in response to disturbed flow. Proc Natl Acad Sci USA 106: 8326–8331. 19416856 10.1073/pnas.0903197106 PMC2676169

[35] Yu C, Minemoto Y, Zhang J, Liu J, Tang F, et al (2004) JNK suppresses apoptosis via phosphorylation of the proapoptotic Bcl-2 family protein BAD. Mol Cell 13: 329–340. 14967141 10.1016/s1097-2765(04)00028-0

[36] Wang Z, Wang H, Xu ZM, Ji YL, Chen YH, et al (2012) Cadmium-induced teratogenicity: association with ROS-mediated endoplasmic reticulum stress in placenta. Toxicol Appl Pharmacol 259: 236–247. 22252055 10.1016/j.taap.2012.01.001

[37] Chen L, Xu B, Liu L, Luo Y, Zhou H, et al (2011) Cadmium induction of reactive oxygen species activates the mTOR pathway, leading to neuronal cell death. Free Radic Biol Med 50: 624–632. 21195169 10.1016/j.freeradbiomed.2010.12.032 PMC3032035

[38] Hetz C, Bernasconi P, Fisher J, Lee AH, Bassik MC, et al (2006) Proapoptotic BAX and BAK modulate the unfolded protein response by a direct interaction with IRE1α. Science 312: 572–576. 16645094 10.1126/science.1123480

[39] Hetz C, Glimcher LH (2009) Fine-tuning of the unfolded protein response: Assembling the IRE1α interactome. Mol Cell 35: 551–561. 19748352 10.1016/j.molcel.2009.08.021 PMC3101568

[40] Klee M, Pallauf K, Alcalá S, Fleischer A, Pimentel-Muiños FX (2009) Mitochondrial apoptosis induced by BH3-only molecules in the exclusive presence of endoplasmic reticular Bak. EMBO J 28: 1757–1768. 19339988 10.1038/emboj.2009.90 PMC2699367

[41] Lam D, Dickens D, Reid EB, Loh SH, Moisoi N, et al (2009) MAP4K3 modulates cell death via the post-transcriptional regulation of BH3-only proteins. Proc Natl Acad Sci USA 106: 11978–11983. 19587239 10.1073/pnas.0900608106 PMC2707271

[42] Lin JH, Li H, Yasumura D, Cohen HR, Zhang C, et al (2007) IRE1 signaling affects cell fate during the unfolded protein response. Science 318: 944–949. 17991856 10.1126/science.1146361 PMC3670588

[43] Rutkowski DT, Kaufman RJ (2007) That which does not kill me makes me stronger: adapting to chronic ER stress. Trends Biochem Sci 32: 469–476. 17920280 10.1016/j.tibs.2007.09.003

